# Parenting styles and Chinese preschoolers' moral sensitivity: domain-specific associations and moderating roles of child gender and age

**DOI:** 10.3389/fpsyg.2026.1903416

**Published:** 2026-08-24

**Authors:** Weiwei Li, Danyi Su, Chris Moore, Robin Curtis, Xihua Zeng

**Affiliations:** 1 College of Education (Normal School), Dongguan University of Technology, Dongguan, China; 2 University of Guangdong Education, Guangzhou, China; 3 Leliu JiangYi Primary School, Guangdong, China; 4 Department of Psychology and Neuroscience, Dalhousie University, Halifax, NS, Canada; 5 The University of British Columbia, Vancouver, BC, Canada; 6 Department of Psychology, School of Public Health, Southern Medical University, Guangzhou, China; 7 Department of Psychiatry, Zhujiang Hospital, Southern Medical University, Guangzhou, China

**Keywords:** child age, child gender, Chinese culture, maternal parenting, moral foundations theory, moral sensitivity, paternal parenting, preschoolers

## Abstract

Parenting is widely regarded as an important factor in children's early moral development. The present study examined the associations between maternal and paternal parenting styles (emotional warmth, overprotection, and rejection) and preschool children's moral sensitivity across the five moral foundation domains (care, fairness, loyalty, authority, and purity) in a Chinese sample, and tested whether child gender and age moderated these associations. Participants were 143 preschoolers aged 3 to 6 years (70 boys, 73 girls) and their parents from six kindergartens in Guangzhou, China. Parents independently completed the short form of the EMBU; children's moral sensitivity was assessed using a scenario-based transgression evaluation task. Regression analyses revealed that parenting associations were concentrated in the loyalty domain: maternal overprotection (β = −0.25, *p* < 0.01) and paternal emotional warmth (β = −0.21, *p* < 0.05) were both negatively associated with loyalty sensitivity. There was no evidence that child gender moderated any parenting–moral sensitivity association. Bayesian model comparison favored the no-moderation model in this sample. No parenting associations emerged for care, fairness, authority, or purity, and child age did not moderate any association. These findings suggest that parenting is associated with early moral development in a domain-specific and culturally situated way, with loyalty sensitivity being especially responsive to family socialization processes in the Chinese context.

## 1 Introduction

The development of moral sensitivity in young children is a foundational component of moral development, and understanding its early roots is important for supporting healthy socialization. The preschool years represent a particularly sensitive period for the formation

of moral sensitivity (Decety et al., 2011). Yet most studies have concentrated on older children and have typically examined either mothers or fathers in isolation. As a result, the joint roles of both parents in children's moral sensitivity across different moral domains remain poorly understood. Addressing this gap is important in non-Western settings such as the Chinese cultural context, where family roles and expectations may diverge from Western models.

Moral sensitivity refers to the ability to recognize moral issues and identify moral behaviors. It often involves automatic, affectively laden responses to situational cues (Decety et al., 2011; Morton et al., 2006; Rest et al., 2000) and is not solely a product of conscious reasoning. Moral sensitivity has identifiable neural and affective underpinnings, enabling individuals to respond intuitively when confronted with violations of fundamental moral standards shaped by cultural and evolutionary forces (Schweigert, 2016). Moral Foundations Theory (MFT; Haidt, 2012) offers a comprehensive framework for conceptualizing these processes. MFT posits that human morality is rooted in several core domains grounded in evolutionary, cross-cultural, and comparative research, and identifies five principal moral foundations: care/harm, which involves compassion and protection of others; fairness/cheating, which emphasizes justice and reciprocity; loyalty/betrayal, which pertains to group allegiance and cohesion; authority/subversion, which concerns respect for social hierarchies; and purity/degradation, which concerns the maintenance of physical and symbolic purity. Emerging evidence suggests that preschoolers can display moral sensitivity by responding appropriately to vignettes across these five domains (Du, 2020; Smetana, 2006).

The family environment, and specifically parenting style, is considered a key factor in the development of moral sensitivity in early childhood. Parenting style is defined as the emotional climate established by parents through their attitudes, behaviors, and communication patterns (Darling and Steinberg, 1993). According to social learning theory, children learn about moral concepts by observing, imitating, and interacting with their parents (Bandura, 1977). The ways in which parents model moral reasoning and respond to children's behavior contribute to the formation of children's moral frameworks. Positive parenting, marked by warmth, consistency, and open dialogue, has been shown to promote moral sensitivity by providing effective role models, encouraging reasoning and discussion, and fostering a secure emotional environment (Carlo et al., 2011; Davidov and Grusec, 2006; Yavuz et al., 2022; Zhou et al., 2002). Conversely, negative parenting styles such as authoritarianism, overprotection, or neglect are associated with diminished opportunities for moral reasoning and weaker moral sensitivity (Hu and Feng, 2022; Walker, 1999). These findings are reinforced by studies on the importance of parental affection and thoughtful communication about moral issues (Boyes and Allen, 1993; Walker and Hennig, 1999).

Despite these advances, the majority of empirical studies have focused primarily on care and fairness. Relatively little attention has been paid to the domains of loyalty, authority, and purity within the MFT framework (Graham et al., 2013; Haidt and Joseph, 2008). One reason is that early developmental research on moral judgment was rooted in the Kohlbergian tradition, which emphasized justice and welfare reasoning and treated loyalty, authority, and purity as conventional rather than moral concerns. As a result, the broader landscape of children's moral development remains incompletely mapped. To address this gap, empirical studies must examine how varying parenting styles relate to children's moral sensitivity across all five domains proposed by MFT.

In addition to parenting style, the gender of the parent may also play an important role in the development of moral sensitivity. Mothers and fathers often serve as distinct sources of socialization (Lamb, 2010), and they tend to interact with their children in different ways. Maternal parenting is frequently characterized by nurturing, emotional support, and sensitivity, which can foster empathy and prosocial behavior (Davidov and Grusec, 2006; Hastings et al., 2007; Singh et al., 2021; Videon, 2005). For instance, Barni et al. (2020) found that maternal moral values were closely linked with adolescents' internalization of moral norms. In contrast, paternal parenting often emphasizes discipline, social rule-teaching, and the transmission of societal expectations (Smollar and Youniss, 1985; Videon, 2005). These paternal behaviors can also affect prosocial behavior and empathy, though generally to a lesser extent than maternal parenting (Davidov and Grusec, 2006; Hastings et al., 2007; Miklikowska et al., 2011; Wong et al., 2022).

These gendered distinctions in parenting may be especially salient in cultural contexts such as China, where traditional values like filial piety and familial hierarchy strongly shape parenting practices (Ho, 1987; Li, 2020). The traditional Confucian family system assigned fathers the role of disciplinarian and moral guide, particularly in the domains of authority, loyalty, and fairness, while mothers were expected to provide nurturance and maintain moral purity. However, contemporary socioeconomic changes have prompted significant shifts. Chinese mothers are now more actively engaged in moral education, and fathers increasingly participate in child-rearing and share childcare responsibilities (Chinese National Health Family Planning Commission, 2016; Liu et al., 2016; Zhou, 2003). Moreover, the one-child policy has further intensified parental investment and involvement (Guo, 2013; Xu et al., 2018). These shifts suggest that the traditional division of moral labor between Chinese mothers and fathers may be less sharply drawn today, although it remains an empirical question how these changes are reflected in young children's moral sensitivity.

Children's gender and age are also critical variables that may moderate the link between parenting style and moral development (Gryczkowski et al., 2018; Janssens and Deković, 1997; Spruijt et al., 2019). Studies have yielded mixed findings regarding whether the impact of parenting differs for boys and girls. Three general patterns have been identified: stronger effects in same-sex parent–child dyads (Dou et al., 2020; Starrels, 1994), stronger effects in opposite-sex dyads (Davidov and Grusec, 2006; Webster et al., 2013), or no gender moderation (Barni et al., 2020; McKee et al., 2007; Russell et al., 1998). Social learning theory posits that children may model the behaviors of their same-sex parent (Bandura, 1977). For example, Niu et al. (2004) observed that Chinese children's helping behavior was more strongly influenced by their same-sex parent. Nevertheless, some evidence points to the significance of opposite-sex dyads. For instance, Gryczkowski et al. (2018) found that paternal parenting was significantly associated with prosocial behavior in girls aged 6–17, and Isley et al. (1996) and Webster et al. (2013) reported similar opposite-sex effects. Still other studies have observed no gender moderation in the impact of harsh parenting on problem behaviors for both boys and girls (McKee et al., 2007). This inconsistency may stem from varying measures of parenting and child outcomes, and underscores the need for further empirical testing. According to social domain theory, moral rules are obligatory, universalizable, and unalterable (Smetana, 1999). If parents enforce moral standards uniformly regardless of the child's gender, one might expect no gender moderation in the parenting–moral sensitivity relationship. This proposition requires further empirical testing.

Age may also shape how parenting relates to moral development (Gryczkowski et al., 2018; Janssens and Deković, 1997; Spruijt et al., 2019). Some studies report that parenting has a greater effect on younger children, and that this effect weakens as children grow and their social worlds widen (Gryczkowski et al., 2018; Janssens and Deković, 1997). As children gain more experience with peers and teachers, parental guidance may play a smaller role (Janssens and Deković, 1997). Other studies report that some parenting practices continue to shape prosocial behavior into adolescence (Houltberg et al., 2016; Wong et al., 2022). Importantly, this evidence comes from older children, often aged 6 to 11 years or older, while direct evidence within the preschool age range (3–6 years) is scarce. For this reason, we treated child age as an exploratory question rather than a directional prediction.

In summary, the role of parenting in preschoolers' moral sensitivity is multifaceted. It is shaped by parenting style, the gender of both parent and child, and the child's developmental stage, all situated within a specific cultural context. A comprehensive understanding of early moral development requires attention to these interacting factors and recognition of the evolving roles of mothers and fathers.

### 1.1 The present study

The present study examines how maternal and paternal parenting styles (emotional warmth, overprotection, rejection) are associated with Chinese preschoolers' moral sensitivity across five MFT domains: care, fairness, loyalty, authority, and purity. It also tests whether child gender and age moderate these associations. Grounded in MFT (Haidt, 2012) and in social learning and social domain perspectives (Bandura, 1977; Smetana, 1999), we propose two hypotheses and one exploratory research question.

**Hypothesis 1a**. Parenting will be linked more closely to the binding foundations (loyalty, authority, and purity) than to the individualizing foundations (care and fairness). MFT posits that moral foundations are differentially elaborated by culture and social learning (Haidt, 2012). The binding foundations rest on group norms and social hierarchy, which are emphasized in collectivist Chinese families, so we expected parenting to matter more for these domains.

**Hypothesis 1b**. Within the binding foundations, emotional warmth will be positively related, and overprotection and rejection will be negatively related, to moral sensitivity.

**Hypothesis 2**. Child gender will not moderate the associations between parenting and moral sensitivity. Social domain theory treats moral rules as obligatory and applied to all children (Smetana, 1999), which implies that parents hold the same moral standards for boys and girls. Because this is a prediction of no difference, we did not rely on non-significant interaction terms alone. We also used Bayesian model comparison to test whether the data favor the model with no moderation (Dienes, 2014; Wagenmakers, 2007).

**Exploratory question on child age**. We did not set a directional hypothesis for age. Direct evidence on age differences within the narrow 3- to 6-year range is scarce, and reports of weaker parenting effects with age come from older children and adolescents (Houltberg et al., 2016; Janssens and Deković, 1997; Wong et al., 2022). We therefore examined child age as an exploratory moderator, asking whether the associations between parenting and moral sensitivity differ across the preschool years.

## 2 Materials and methods

### 2.1 Participants

We recruited 152 families from six kindergartens in Guangzhou, China. From this pool, we excluded six children who were missing data on the moral sensitivity task, and three children for whom one parent's EMBU report was incomplete, giving nine exclusions in total. The final sample comprised 143 preschoolers aged 3 to 6 years (70 boys) with complete data from the mother, the father, and the child. The sample included children from the junior (*n* = 28; 15 boys; *Mage* = 3.25, *SD* = 0.44), middle (*n* = 44; 22 boys; *Mage* = 4.32, *SD* = 0.47), and senior (*n* = 71; 33 boys; *Mage* = 5.27, *SD* = 0.45) classes. Mothers and fathers were invited to independently complete the parenting questionnaires.

### 2.2 Procedure

Children were tested during one-on-one sessions with an experimenter. The order of the tasks was counterbalanced between participants to control for order effects. Father and mother separately completed a parenting questionnaire to ensure that maternal and paternal parenting were independently captured. Before the formal testing, informed consent was obtained from the kindergarten principals, teachers, and parents. Approval was obtained from the Academic Committee of Guangdong University of Education. The study was conducted in accordance with the Declaration of Helsinki and applicable national ethical guidelines.

### 2.3 Instruments

#### 2.3.1 Children's moral sensitivity across five foundations

Children's moral sensitivity across the five foundation domains was assessed using a scenario-based transgression evaluation task designed for preschool-aged children. The task was based on Moral Foundations Theory (Haidt, 2012) and adapted from the Moral Foundations Vignettes (Clifford et al., 2015). Because preschoolers' language comprehension is still limited, the original text-based vignettes could not be administered directly. We therefore selected vignettes relevant to preschoolers' everyday life and kindergarten settings, adapted the wording to suit their developmental level, and presented each scenario with simple stick-figure sticker illustrations.

The task included 20 short vignettes, four in each domain. Each vignette described a concrete transgression: care (for example, harm to a person or an animal), fairness (for example, unfair treatment in a game), loyalty (for example, betraying a familiar group or trusted relationship, such as revealing another person's secret or helping the opposing team), authority (for example, disobeying a teacher or a parent), and purity (for example, contact with something dirty or disgusting). For preschoolers, loyalty was framed around concrete, familiar relationships and groups (such as peers, classmates, or siblings), not abstract entities such as a nation or institution. Each vignette described actions and outcomes only. It did not use evaluative words such as fair, good, bad, or obedient, so that the child's rating, and not the wording, carried the moral sensitivity. After each vignette, children were asked how wrong the act was and rated their response on a 5-point pictorial scale, from 1 (*not wrong at all*) to 5 (*very wrong*). All vignettes were read aloud in simple language with colorful pictures. A comprehension check followed each vignette, and trials in which the child could not retell the core event were dropped. A similar vignette-based approach has been used in earlier studies of young children's moral evaluation (Smetana, 2006). An example vignette and the full set of 20 vignettes are provided in Supplementary Appendix.

In the present sample, internal consistency varied across the five subscales: care (α = 0.63, ω = 0.65), fairness (α = 0.50, ω = 0.57), loyalty (α = 0.52, ω = 0.54), authority (α = 0.62, ω = 0.64), and purity (α = 0.70, ω = 0.73). Two subscales, fairness and loyalty, fell below the common 0.70 threshold. Because alpha depends on the number of items, we also examined item-level indices that are recommended when alpha is low (Clark and Watson, 1995; Tavakol and Dennick, 2011). Mean inter-item correlations ranged from 0.20 (fairness) to 0.39 (purity), within the 0.15 to 0.50 range that Clark and Watson (1995) regard as acceptable, and corrected item-total correlations were all positive (range 0.19 to 0.58). These indices indicate that each four-item subscale is internally coherent and that the low alpha values mainly reflect the small number of items. Robustness checks for the loyalty findings, using factor scores and disattenuation, are reported in the Results.

#### 2.3.2 The revised Chinese-EMBU

Parenting styles were assessed with the Chinese short form of the EMBU (Perris et al., 1980; Zhang, 2010), validated for parents of young children. Mothers and fathers completed the measure independently. Three subscales were included: emotional warmth (12 items; e.g., “I show affection to my child through hugging or praising”), overprotection (8 items; e.g., “I worry a great deal about my child's safety and limit his/her activities”), and rejection (8 items; e.g., “I often tell my child that I do not like his/her behavior at home.”). We retain the EMBU label “rejection” to stay comparable with the original instrument. This subscale captures rejecting parenting that is directed at the child, such as criticism and expressions of disapproval. Items were rated on a 4-point scale from 1 (*never*) to 4 (*always*). Internal consistency in the present sample was adequate to good. For maternal scales, Cronbach's α values were 0.89 (emotional warmth), 0.72 (rejection), and 0.76 (overprotection). For paternal scales, the corresponding α values were 0.90, 0.70, and 0.77.

#### 2.3.3 Data analysis

Before testing the hypotheses, we computed descriptive statistics and correlations for all variables (see Table 1). Analyses were run in SPSS Statistics 25.0. First, we used separate regression analyses to examine the associations of maternal and paternal parenting with preschoolers' moral sensitivity (see Table 2). Each parenting dimension was entered as the only predictor in its own model, so each main-effect coefficient is a single-predictor standardized coefficient and equals the corresponding zero-order correlation. We did not enter the parenting dimensions together, to avoid collinearity between the maternal and paternal scales. Second, to test whether child gender moderated these associations across the five domains, we used hierarchical regression. In the first step, a parenting variable (for example, maternal emotional warmth) and child gender were entered as predictors of one moral domain. In the second step, the parenting by gender interaction term was added. We used the same models to test moderation by child age. All continuous variables were mean-centered before the interaction terms were computed. Because Hypothesis 2 is a prediction of no moderation, we tested it with Bayesian model comparison, using the BIC approximation to the Bayes factor (Kass and Raftery, 1995; Wagenmakers, 2007). This weighs the evidence for the no-moderation model rather than relying on non-significant interaction terms alone (Dienes, 2014). We also ran a sensitivity power analysis in G^*^Power 3.1 (Faul et al., 2009), and we computed McDonald's ω in addition to Cronbach's alpha (Hayes and Coutts, 2020). These analyses were reproduced in R 4.3, and the de-identified data and analysis scripts are available from the corresponding author on request.

**Table 1:** Descriptive statistics and correlations (*N* = 143).

Variable	M	SD	1	2	3	4	5	6	7	8	9	10	11
1. Maternal warmth	3.32	0.39	—										
2. Paternal warmth	3.20	0.43	0.18^*^	—									
3. Maternal overprotection	2.05	0.29	0.07	0.08	—								
4. Paternal overprotection	1.88	0.34	−0.18^*^	−0.24^**^	0.22^**^	—							
5. Maternal rejection	1.36	0.28	−0.41^***^	−0.07	0.35^***^	0.26^**^	—						
6. Paternal rejection	1.37	0.32	−0.18^*^	−0.33^***^	0.01	0.59^***^	0.22^**^	—					
7. Care	4.15	0.79	0.01	−0.04	−0.07	v0.09	0.06	−0.01	—				
8. Fairness	3.84	0.81	−0.03	−0.04	−0.11	−0.11	0.03	−0.07	0.54^***^	—			
9. Authority	3.98	0.80	−0.00	0.03	0.01	−0.00	0.12	−0.01	0.61^***^	0.65^***^	—		
10. Loyalty	3.58	0.88	−0.09	−0.21^*^	−0.25^**^	−0.01	0.00	0.03	0.47^***^	0.62^***^	0.52^***^	—	
11. Purity	4.11	0.86	0.02	−0.02	−0.02	−0.05	0.10	0.01	0.63^***^	0.60^***^	0.65^***^	0.51^***^	—

^*^p < 0.05; ^**^p < 0.01; ^***^p < 0.001.

**Table 2:** Regression coefficients for parenting styles and the moderating roles of child gender and age in preschoolers' moral sensitivity across five moral foundation domains [β (*SE*)].

Predictor	Care	Fairness	Authority	Loyalty	Purity
Main effects					
Mwarm	0.01 (0.08)	−0.03 (0.08)	−0.00 (0.08)	−0.09 (0.08)	0.02 (0.08)
Pwarm	−0.04 (0.08)	−0.04 (0.08)	0.03 (0.08)	**−0.21** ^*^ **(0.08)**	−0.02 (0.08)
Moverprotection	−0.07 (0.08)	−0.11 (0.08)	0.01 (0.08)	**−0.25** ^**^ **(0.08)**	−0.02 (0.08)
Poverprotection	−0.09 (0.08)	−0.11 (0.08)	−0.00 (0.08)	−0.01 (0.08)	−0.05 (0.08)
Mrejection	0.06 (0.08)	0.03 (0.08)	0.12 (0.08)	0.00 (0.08)	0.10 (0.08)
Prejection	−0.01 (0.08)	−0.07 (0.08)	−0.01 (0.08)	0.03 (0.08)	0.01 (0.08)
Child gender	−0.13 (0.08)	0.07 (0.08)	−0.02 (0.08)	0.07 (0.08)	−0.09 (0.08)
Child age	0.19^*^ (0.08)	−0.03 (0.08)	0.07 (0.08)	−0.06 (0.08)	0.08 (0.08)
×Child gender interactions					
Mwarm x gender	0.03 (0.08)	−0.03 (0.08)	0.00 (0.09)	0.04 (0.08)	−0.15 (0.08)
Pwarm x gender	−0.12 (0.09)	−0.13 (0.09)	−0.11 (0.09)	−0.10 (0.08)	−0.16 (0.08)
Moverprotection x gender	−0.11 (0.08)	−0.09 (0.08)	−0.09 (0.09)	−0.02 (0.08)	−0.15 (0.08)
Poverprotectionx gender	0.04 (0.08)	0.10 (0.08)	0.06 (0.09)	0.02 (0.08)	−0.03 (0.08)
Mrejection x gender	−0.12 (0.08)	−0.13 (0.08)	−0.11 (0.08)	−0.14 (0.08)	−0.14 (0.08)
Prejection x gender	0.09 (0.09)	0.03 (0.09)	0.04 (0.09)	−0.09 (0.09)	0.04 (0.09)
×Child age interactions					
Mwarm x age	0.01 (0.08)	0.04 (0.08)	0.04 (0.08)	−0.05 (0.08)	−0.00 (0.08)
Pwarm x age	0.08 (0.08)	−0.02 (0.08)	−0.01 (0.08)	−0.11 (0.08)	−0.04 (0.08)
Moverprotection x age	−0.03 (0.09)	−0.00 (0.09)	−0.08 (0.09)	0.03 (0.09)	−0.05 (0.09)
Poverprotection x age	−0.03 (0.09)	−0.01 (0.10)	−0.06 (0.10)	−0.00 (0.10)	−0.05 (0.10)
Mrejection x age	−0.09 (0.08)	−0.05 (0.08)	−0.11 (0.08)	−0.00 (0.08)	−0.09 (0.08)
Prejection x age	−0.03 (0.09)	0.06 (0.09)	0.02 (0.09)	0.12 (0.09)	0.03 (0.09)

Mwarmth, maternal emotion warmth: Pwarmth, paternal emotion warmth; Moverprotection, maternal overprotection; Poverprotection, paternal overprotection; Mrejection, maternal rejection; Prejection, paternal rejection. ^*^p < 0.05; ^**^p < 0.01. Child gender was coded boys = 0, girls = 1. Main effects are from separate single-predictor regressions. Each interaction is from a model containing that parenting dimension, the moderator, and their product. *N* = 143. The bold values represent significance.

## 3 Results

### 3.1 Associations of maternal and paternal parenting with preschoolers' moral sensitivity

Two parenting dimensions were associated with children's moral sensitivity. Maternal overprotection was negatively associated with children's loyalty sensitivity (β = −0.25, *p* < 0.01), indicating that children whose mothers reported more overprotective parenting judged loyalty transgressions as less severe. Paternal emotional warmth was also negatively associated with loyalty sensitivity (β = −0.21, *p* < 0.05), indicating that children whose fathers reported more emotional warmth showed lower loyalty sensitivity. No other parenting dimension was associated with moral sensitivity in any of the five domains (all *p*s >0.05). Child age was positively associated with care sensitivity (β = 0.19, *p* < 0.05), suggesting that older preschoolers rated care transgressions as more severe. The two loyalty associations held when we used factor scores (β = −0.22 for maternal overprotection and β = −0.23 for paternal warmth). They also held when we corrected the two loyalty correlations for the modest reliability of the loyalty subscale. We divided each correlation by the square root of its reliability (Spearman, 1904). The corrected *r*s were −0.35 and −0.29. We corrected only for the outcome measure, because the parenting scales were reliable (α = 0.76 to 0.90). In both checks the associations stayed negative and similar in size or larger, so they are not an artifact of low reliability.

### 3.2 Moderation of child's gender and age on parenting and preschoolers' moral sensitivity

No parenting by child gender interaction was significant in any domain (Table 2). Bayesian model comparison supported the absence of moderation. In 27 of the 30 interaction tests, the data favored the model with no moderation by more than a factor of three (*BF*_01_ > 3), and no test favored moderation (Dienes, 2014; Kass and Raftery, 1995). The paternal warmth by gender term for purity was not significant (β = −0.16, *p* >0.05), and its Bayesian evidence was weak and inconclusive (*BF*_01_ = 1.89).

With respect to child age, although the main effect on care was significant (β = 0.19, *p* < 0.05), no significant age-by-parenting interaction effects were observed for any of the five moral domains (see Table 2). As a result, further multiple-group regression analyses by age were not warranted.

## 4 Discussion

This study examined how maternal and paternal parenting styles related to Chinese preschoolers' moral sensitivity across the five moral foundation domains, and whether child gender and age moderated these associations. Three findings emerged. First, parenting was associated with moral sensitivity only in the loyalty domain. Second, we did not detect moderation by child gender in any association. Bayesian model comparison favored the no-moderation model. Third, child age did not moderate any association, although older preschoolers showed higher care sensitivity.

### 4.1 Domain-specific parenting–loyalty associations

Hypothesis 1 predicted that parenting would relate more closely to the binding foundations (loyalty, authority, purity) than to the individualizing foundations (care, fairness). The results partially supported this hypothesis. Among the binding foundations, only loyalty was associated with parenting. The individualizing foundations showed none. Maternal overprotection was negatively related to loyalty sensitivity, which matches the direction predicted for a negative parenting dimension. Paternal emotional warmth was also negatively related to loyalty sensitivity, which was contrary to our prediction. These associations held when we used factor scores and when we corrected for measurement error, indicating that they are unlikely to be artifacts of low subscale reliability.

This domain-specific pattern is consistent with MFT's claim that moral foundations are culturally elaborated to different degrees (Haidt, 2012). Confucian socialization places strong emphasis on collective harmony, filial piety, and in-group solidarity (Ho, 1987; Hwang, 1999). Loyalty-related norms are therefore a salient focus of Chinese family-based moral socialization. To the extent that loyalty norms are more actively transmitted within the family than norms governing harm or fairness, individual differences in parenting should be more strongly reflected in loyalty sensitivity than in the other domains. Care and fairness sensitivity, by contrast, may depend more on maturational processes and on peer experience during the preschool years (Smetana, 2006). The positive association between child age and care sensitivity is consistent with this interpretation.

The inverse association between maternal overprotection and loyalty sensitivity is consistent with theoretical expectations. Overprotective parenting restricts children's autonomous peer engagement, including group play and cooperative tasks (Young et al., 2013). These experiences are central to the formation of in-group identification. By restricting the child's peer interactions, overprotective mothers may reduce the experiences from which loyalty intuitions emerge.

The inverse association between paternal emotional warmth and loyalty sensitivity was contrary to our prediction. Warmth is generally linked to better moral outcomes in Western samples (Davidov and Grusec, 2006; Zhou et al., 2002). One possible explanation draws on the contemporary urban Chinese context. Highly warm and child-centered fathering, sometimes described as the “cat father” pattern (in contrast to the stricter “tiger father” style; Guo, 2013; Li, 2020), may emphasize the child's individual emotional experience over obligations to the in-group. This interpretation is consistent with the distinction between parenting style and parenting practices (Darling and Steinberg, 1993). A warm relational climate may support general wellbeing without conveying specific moral content, such as duty to the in-group, that requires explicit teaching or modeling. This explanation is *post hoc* and was not tested directly. It generates a testable prediction: the paternal warmth–loyalty association should attenuate or reverse when warmth is measured alongside paternal practices that explicitly convey collectivist norms. Studies that disaggregate warmth from the specific moral content of warm interactions would be needed to test this explanation.

The absence of parenting associations in care, fairness, authority, and purity deserves comment. One possibility is developmental: across ages 3–6, sensitivity in these domains may depend more on maturation and on contact with peers and teachers than on parenting style. The age–care association supports this possibility. A second possibility is cultural. In Confucian socialization, obedience to elders is required of all children (Hwang, 1999). This uniform pressure may produce ceiling effects in authority sensitivity and leave little variance for parenting to explain. A third possibility is methodological. The modest reliability of two subscales and the limited sample size may have prevented small true effects from reaching significance. For these reasons, the four domains are best described as showing no detected effect rather than no effect.

### 4.2 Child gender did not moderate parenting–moral sensitivity associations

Hypothesis 2 predicted no gender moderation, based on social-domain theory, which holds that moral rules are obligatory and applied to all children (Smetana, 1999). The results were consistent with this prediction. No parenting × child gender interaction reached significance in any of the five domains. Bayesian model comparison provided positive evidence for the no-moderation model in 27 of 30 tests (BF01 > 3), with the remaining three tests inconclusive and none favoring moderation. Because Hypothesis 2 is a null prediction, this Bayesian evidence is essential: it distinguishes evidence of absence from absence of evidence (Altman and Bland, 1995; Dienes, 2014).

These results speak directly to the mixed literature on child gender. Some studies report stronger effects in same-sex dyads (Dou et al., 2020; Starrels, 1994), and others report stronger effects in opposite-sex dyads (Davidov and Grusec, 2006; Gryczkowski et al., 2018; Webster et al., 2013). Our data align with the third pattern, namely no gender moderation (Barni et al., 2020; McKee et al., 2007; Russell et al., 1998). The convergence of frequentist and Bayesian null results across all five moral domains suggests that, in the Chinese preschool context, parents apply moral standards to boys and girls in broadly similar ways. This conclusion is limited by sample size; replication in larger and more diverse samples is needed before the absence of gender moderation can be regarded as established.

### 4.3 Child age did not moderate parenting–moral sensitivity associations

Child age did not moderate any parenting association. Earlier studies have reported weaker parenting effects with age (Gryczkowski et al., 2018; Janssens and Deković, 1997), but those studies covered much broader age spans extending into adolescence. The present sample covered only ages 3–6. Across this narrow range, parents remain the primary socialization agents, and children's social experiences outside the family are limited. Age may begin to differentiate parenting associations only over a wider developmental span, a question that longitudinal designs extending into middle childhood would be well suited to address.

### 4.4 Contributions and limitations

The study makes three contributions. First, it provides early evidence that parenting relates to preschoolers' moral sensitivity in a domain-specific rather than a domain-general way. Loyalty was the foundation most sensitive to variation in parenting in the Chinese context, consistent with MFT's view that culture shapes the elaboration of moral foundations (Haidt, 2012). Second, the distinct roles of maternal overprotection and paternal emotional warmth show the value of treating mothers and fathers as separate sources of socialization rather than as a single unit (Lamb, 2010). Third, the study asks whether parenting relates to moral sensitivity differently for boys and girls. Some studies report gender-differentiated parenting effects, including early in childhood (Dou et al., 2020; Gryczkowski et al., 2018; Webster et al., 2013). We did not find this pattern. Child gender did not moderate any parenting association across the five domains. However, a non-significant result alone cannot support a claim of no moderation. We therefore used Bayesian model comparison, which favored the no-moderation model (Dienes, 2014; Wagenmakers, 2007). This gives positive evidence for the absence of moderation in our sample. It does not prove that gender never matters.

Several limitations should be noted. The sample was drawn from a single city, which limits generalizability. The sample size (*N* = 143) afforded adequate power for medium effects but limited power for small ones. A sensitivity power analysis in G^*^Power 3.1 (Faul et al., 2009) indicated that, with one predictor entered at the final step, the study had 1–β = 0.80 to detect effects of *f* ^2^ ≥ 0.056 (equivalent to a partial correlation of *r* = 0.23). Power was high for medium effects (1–β = 0.996 at *f* ^2^ = 0.15) but low for small effects (1–β = 0.39 at *f* ^2^ =0.02; Cohen, 1988). Detecting a small effect at 80% power would require approximately *N* = 395. Non-significant frequentist results should therefore be interpreted as undetected effects rather than as evidence of absence; the Bayesian analyses partly mitigate this concern for the gender-moderation tests. Two subscales (fairness, loyalty) showed modest internal consistency, which we addressed with item-level diagnostics and with factor-score and disattenuation robustness checks; this issue indicates the need for improved measures in future work. The design is cross-sectional and therefore does not support causal claims. Parent self-reports may carry some social desirability bias. Future studies would benefit from larger and more diverse samples, longitudinal designs, observational assessments of parenting behavior, and replication in other cultural contexts.

## 5 Conclusion

In conclusion, parenting was associated with Chinese preschoolers' moral sensitivity in a domain-specific way, with associations concentrated in the loyalty domain. Maternal overprotection and paternal emotional warmth were each negatively associated with loyalty sensitivity. Child gender did not emerge as a moderator of these associations. No interaction was significant, and Bayesian model comparison favored the no-moderation model. In our data, then, parenting related to moral sensitivity in similar ways for boys and girls. This result does not rule out gender differences. A larger or more diverse sample might detect differences that ours could not. The findings underscore the value of examining mothers and fathers separately, attending to cultural context, and treating a carefully tested null result as informative when studying the early family roots of moral development.

## Data Availability

The raw data supporting the conclusions of this article will be made available by the authors, without undue reservation.
