# Supplementary material for: Parenting styles and Chinese preschoolers' moral sensitivity: domain-specific associations and moderating roles of child gender and age
Source: Front Psychol. 2026 Aug 24;17:1903416. doi: 10.3389/fpsyg.2026.1903416 (PMC13546979; doi:10.3389/fpsyg.2026.1903416)
Supplement: Supplementary file 1 [file Table_1.docx]

Supplementary Appendix

(Preschoolers’ Moral Sensitivity Across Five Moral Foundation Domains Measurement)

Children’s moral sensitivity across the five foundation domains was assessed using a scenario-based transgression evaluation task designed for preschool-aged children. The task was based on Moral Foundations Theory (Haidt, 2012) and adapted from the Moral Foundations Vignettes (Clifford et al., 2015). Because preschoolers’ language comprehension is still developing, the original text-based vignettes were adapted into age-appropriate scenarios depicting everyday experiences in kindergarten and family settings. Each vignette was presented with simple stick-figure illustrations while the experimenter read the scenario aloud. After each vignette, children were asked: "How wrong was what this child did?" Responses were made using a 5-point pictorial scale ranging from 1 (Not wrong at all) to 5 (Very wrong). The task comprised 20 vignettes, with four vignettes representing each moral foundation.

## Care (Items 1–4)

1. You see a child stepping hard on the tail of his or her pet cat. (Chinese: 你看见一个小朋友用力踩自己宠物猫的尾巴。)

**2.** You see a child punching another child in the stomach. (Chinese:你看见一个小朋友打另外一个小朋友的肚子。)

**3.** You see a child kicking a stray dog. (Chinese:你看见一个小朋友踢一条流浪狗。)

4. You see a child calling another child “idiot.” (Chinese: 你看见一个小朋友叫另外一个小朋友“笨蛋”。)

## Fairness (Items 5–8)

5. You see a child cutting in line instead of waiting. (Chinese: 你看见一个小朋友排队时插队。)

6. You see a child taking all the cookies and leaving none for others. (Chinese:你看见一个小朋友拿走所有的饼干，一个都没有留给别人。)

7. You see a child taking a shortcut to cheat during a running race. (Chinese: 你看见一个小朋友在跑步比赛中走小路作弊。)

8. You see a child cheating during a card game. (Chinese: 你看见一个小朋友在扑克牌游戏中作弊。)

## Loyalty (Items 9–12)

9. You see a child telling his or her brother’s secret to someone the brother dislikes. (Chinese: 你看见一个小朋友把哥哥的秘密告诉哥哥不喜欢的人。)

10. You see a child secretly telling another class about his or her own class’s secret. (Chinese: 你看见一个小朋友把班级的秘密偷偷告诉其他班级的人。)

11. You see a child deliberately kicking the ball into his or her own team’s goal to help the opposing team win. (Chinese: 你看见一个小朋友把球踢进自己球队的门框，帮助对手获胜。)

12. You see a child secretly reading his or her brother’s diary. (Chinese: 你看见一个小朋友偷偷看哥哥的日记。)

**Authority (Items 13–16)**

13. You see a child swearing at his or her parents. (Chinese: 你看见一个小朋友对父母说脏话。)

14. You see a child swearing at the teacher. (Chinese: 你看见一个小朋友对老师说脏话。)

15. You see a child continuing to watch television after being told by his or her parents to stop. (Chinese: 你看见一个小朋友在父母不让看电视的时候，继续看电视。)

16. You see a child continuing to wear a hat after the teacher has told everyone not to wear hats. (Chinese:你看见一个小朋友在老师不让带帽子的时候，继续戴帽子。)

## Purity (Items 17–20)

17. You see a child using a dirty diaper as a pillow. (Chinese: 你看见一个小朋友把脏尿布当做枕头。)

18. You see a child rubbing feces on his or her body while taking a bath. (Chinese: 你看见一个小朋友在洗澡时把粪便擦在自己身上。)

19. You see a child drinking urine during dinner. (Chinese: 你看见一个小朋友在晚餐时喝尿。)

20. You see a child deliberately burping and farting loudly during a meal. (Chinese: 你看见一个小朋友在吃饭时发出很大的打嗝和放屁的声音。)
